# Laparoscopic *vs*. open approach for colorectal cancer: evolution over time of minimal invasive surgery

**DOI:** 10.1186/1471-2482-13-S2-S12

**Published:** 2013-10-08

**Authors:** Antonio Biondi, Giuseppe Grosso, Antonio Mistretta, Stefano Marventano, Chiara Toscano, Filippo Drago, Santi Gangi, Francesco Basile

**Affiliations:** 1 Department of General Surgery, Section of General Surgery and Oncology, University Medical School of Catania, Italy; 2 Department of Drug Sciences, Section of Biochemistry, University of Catania, Catania, Italy; 3 Department "G. F. Ingrassia" Section of Hygiene and Public Health, University of Catania, Catania, Italy; 4 Department of Clinical and Molecular Biomedicine, Section of Pharmacology and Biochemistry, University of Catania, Catania, Italy

## Abstract

**Background:**

In the late '80s the successes of the laparoscopic surgery for gallbladder disease laid the foundations on the modern use of this surgical technique in a variety of diseases. In the last 20 years, laparoscopic colorectal surgery had become a popular treatment option for colorectal cancer patients.

**Discussion:**

Many studies emphasized on the benefits stating the significant advantages of the laparoscopic approach compared with the open surgery of reduced blood loss, early return of intestinal motility, lower overall morbidity, and shorter duration of hospital stay, leading to a general agreement on laparoscopic surgery as an alternative to conventional open surgery for colon cancer. The reduced hospital stay may also decrease the cost of the laparoscopic surgery for colorectal cancer, despite th higher operative spending compared with open surgery. The average reduction in total direct costs is difficult to define due to the increasing cost over time, making challenging the comparisons between studies conducted during a time range of more than 10 years. However, despite the theoretical advantages of laparoscopic surgery, it is still not considered the standard treatment for colorectal cancer patients due to technical limitations or the characteristics of the patients that may affect short and long term outcomes.

**Conclusions:**

The laparoscopic approach to colectomy is slowly gaining acceptance for the management of colorectal pathology. Laparoscopic surgery for colon cancer demonstrates better short-term outcome, oncologic safety, and equivalent long-term outcome of open surgery. For rectal cancer, laparoscopic technique can be more complex depending on the tumor location. The advantages of minimally invasive surgery may translate better care quality for oncological patients and lead to increased cost saving through the introduction of active enhanced recovery programs which are likely cost-effective from the perspective of the hospital health-care providers.

## Background

Despite the decreased incidence rates reported during last years, cancer remain the leading cause of death worldwide [1]. It has been reported that only a small part of cancers is genetically determined, and most of them is due to a biological response to environmental factors [2-6]. Interventions focused on primary prevention mostly regard tobacco smoking, alcohol consumption, and dietary advices [7,8], but the burden of the disease is far to be considered negligible. Regarding the therapeutic approaches, in the late '80s the successes of the laparoscopic surgery for gallbladder disease laid the foundations on the modern use of this surgical technique in a variety of diseases [9-14]. Among the most frequent benign and malignant disease which require a surgical therapy, colorectal cancer has reached the best results with a laparoscopic approach in terms of safety [15], reduced postoperative recovery [16], and improved long-term survival [17,18].

Thus, in the last 20 years, laparoscopic colorectal surgery had become a popular treatment option for colorectal cancer patients. Several clinical trials emphasized the aforementioned benefits stating the significant advantages of reduced blood loss, early return of intestinal motility, lower overall morbidity, and shorter duration of hospital stay in the laparoscopic-assisted group, leading to a general agreement on laparoscopic surgery as an alternative to conventional open surgery for colon cancer. However, despite the theoretical advantages of laparoscopic surgery, it is still not considered the standard treatment for colorectal cancer patients due to technical limitations or characteristics of the patients that may affect short and long term outcomes [19]. Thus, the aim of this study is to review the main available evidences between the conventional open approach and laparoscopic resection of colorectal cancer treatment.

## Operative parameters

Results about mean operating time of the laparoscopic-assisted procedure versus open surgery vary among studies, some reporting no significant differences between the two groups [20,21] and others reporting a longer time for the laparoscopic-assisted procedure [22].

The prolonged operative time for the laparoscopic procedure depend on the higher complexity of technical expertise involved in such technique. Given the technical difficulty of this treatment, reasons of such results may be depended by the need for experienced surgeons and a not well established manuality due to a consistent learning curve [23,24]. Major difficulties of the laparoscopic colorectal surgery are due to work in multiple abdominal quadrants, control of vascular structures, creation of anastomosis, as well as retrieving large specimens in some patients whereas potential risks regard port-site recurrence after curative resection of tumor and incomplete lymph node dissection [25,26]. More recent studies are reporting less significant differences according to this parameter thanks to the stabilization of the learning curve of the surgeon. Indeed, in most of the reports, the learning curve of the technique is incorporated during the study period and the skills were still evolving during the conduct of the study, thus is not surprising that as time passed, the surgeon's experience with the procedure increased as well, leading to a decrease of the operative time in the latter phase of the study period.

Blood loss and analgesic requirement depend greatly on the degree of invasiveness of the surgical approach. Results of a recent meta-analysis of clinical trials revealed a significant increased reduction of intraoperative blood loss, number of blood transfusions, and abdominal bleeding in patients who underwent a laparoscopic colon resection compared with those operated with an open approach [27].

## Short-term outcomes

Early randomized controlled trials suggested that the short-term outcomes of laparoscopic colorectal surgeries were similar or barely better than the traditional open approach.

With the establishment of the technique as a routine surgical approach, a study conducted in 48 institutions (namely including many surgeons) on 872 patients, reported that a greater experience of the surgeon (20 or more laparoscopic resections) was associated with longer operating time, but better outcomes such as lower intraoperative complications and shorter recovery time and hospital stay [28].

The significant improvements in postoperative recovery among laparoscopic-treated patients regard mostly an earlier resumption of normal diet, shorter hospital stay, and earlier time to ambulation [20].

However, comparison of length of hospital stay after surgery among studies may be affected by some confounding factors. For instance, socioeconomic status may lead to health care disparities in countries such as the US where insurance play an important role on health care of population. Thus, compared with countries in which the healthcare system provides ensuring equity in the availability of care by removing financial barriers for all patients, the hospital stay of colorectal cancer patients living in US has been reported to be shorter [29]. Taking into account such possible bias, a country-specific comparison of the length of hospital stay of the patients operated with the laparoscopic and open procedure still remain significantly different. Indeed, the postoperative hospital stay for patients who undergo the laparoscopic procedure has been reported to range between about 5 and 7 days in US [28,30,31] and slightly longer in countries in which care are free of charge for all patients [20], compared with 8 to 10 days for those who undergo open surgery.

The benefits in short-term outcomes with laparoscopic resection has been also supported by the reports on perioperative immunologic response [32,33]. In the early post-operative period, better reserved cellular immune responses such as higher levels of total lymphocytes, CD4 T cell, and CD8 T cell in laparoscopic resection compared with open resection was observed [34]. In some studies exploring the inflammatory response differences among different surgical techniques, the immunologically beneficial IFN-γ, produced by the principal effectors of cell-mediated immunity Th1 cells, seemed to have a more active presence following laparoscopic colectomy, potentially contributing to an immunologic "advantage" by counteracting "harmful" cytokines, such as IL-1 [35,36]. Better preserved inflammatory function after surgery may reflect a reduction in operative trauma when the laparoscopic technique is compared with open procedures.

## Long-term outcomes

Body's immunologic function, especially cellular immunity, play a central role in preventing cancer recurrence immediately after surgery of oncological patients [37]. Since the laparoscopic approach limits the tissue trauma and lead to significant less physiologic alterations during the perioperative period, it has been hypothesized that this increased preservation of the immunologic function may be translated in better long-term oncologic outcomes and may be correlated with higher postoperative survival rates [38-40]. Although evidence from early basic science studies seems to be promising, results from human studies are contrasting.

In terms of overall survival, disease-free survival, local or distant recurrence, and long-term quality of life for colon cancer, recent trials results did not show difference between the two groups, laparoscopic and laparotomy resection [41].

Others multicentre and randomized trials have extended patient recruitment including individuals affected by rectal cancer, considering the same parameters [30,42,43].

Taking into consideration the same data, some authors have performed some meta-analyses reporting generally equal long term outcomes [34,44,45]. Oncological safety of patients operated with laparoscopic approach strongly depends on the experience of the surgeon, that has been shown to lead to better long-term outcomes, even when comparing laparoscopic to open surgery [28,30,42]. The studies pointed out also the risk connected with conversion rates, which are indeed reduced by increasing the experience of the surgeon. Some reports suggested that the conversion to open does not affect the general patient long-term outcomes [46], whereas others have shown an increase in the morbidity of inpatients and related outcomes [47-49]. In most cases the conversion depends on advanced cancer stage or, among the most reported reasons, on technical difficulties, obesity, and intra-operatory complications, but since the adverse impact of conversion has been reported to affect the overall survival (and not the disease-free survival), this finding is not attributable to a surgeon-related factor [41].

The problem with incision site recurrences is certainly not a new issue in surgical oncology, but it remains unclear whether if laparoscopy is significantly affected by this issue and what are the mechanisms responsible [50]. For this reason, during the operation, manipulation of the tumor must be avoided; this is possible thanks to the use of non-traumatic forceps but above all thanks to the technique used that provides the fixity of the intestine during the entire surgery, only at the end, its mobilization. The most risky moment is the opening of the abdominal portion for the extraction of the removed piece, during which also happens the deflating of the pneumoperitoneum. The rapid deflation of the pneumoperitoneum may determine the so called "chimney effect" consequently with neoplastic parietal dissemination and the trocar sites. These two events can be avoided using small steps such as the use of endobag and a gradual desufflation with a laparoscopic extractor fan. The volume and extension of the mass are elements that affect contamination of surgical instruments during surgery and the consequential dissemination of neoplastic cells because, when the mass grows on the intestinal serosa or has infiltrated the mesentery and the surrounding structures, contamination becomes inevitable [51-57].

Previous trials reported higher recurrence rates among laparoscopic operated patients compared with those operated with the open approach (up to 80% within 12 months), but latest updates from large randomized control trials do not confirm such rates, reporting comparable results between the two techniques (ranging from 0.5% and 1.3%) [31,54,58].

Incisional hernia and adhesions are also a cause of postoperative morbidity and predictors of long term adverse outcome. Regarding incisional hernia, it has been reported that laparoscopic technique may have some advantages compared with open surgery [59-61], due to the absence of a large abdominal wound [62,63]. Moreover, some authors have reported lower rates of formation of adhesions and related complications in the laparoscopic compared with open group [64-68].

## Cost analysis

Despite the laparoscopic approach has proved as useful for many benign conditions, including diverticulitis, Crohn's disease, and rectal prolapse, the cost-effectiveness and long-term outcomes for malignancy are less well accepted. Earlier studies comparing costs from colectomy by the laparoscopic and open approaches reported conflicting results. Some studies reported costs for laparoscopic colectomy to be greater than for open surgery [69,70] mostly due to higher operative spending for laparoscopy, rising the doubt that the laparoscopic colorectal resection could be potentially less cost-effective than open surgery. Indeed, the care of patients undergoing colorectal surgery is associated with a variety of direct costs related to the operating equipment and consumables, anesthesia, laboratory, radiology, and pharmacy. The laparoscopic approach has been demonstrated that require greater costs related to longer operative time and more expensive equipment. On the other hand, studies started to focus on many other aspects of the comparison between the two techniques, analyzing whether laparoscopic operating room costs were balanced by postoperative care savings [71]. Indeed, compared with conventional open resections, laparoscopic colorectal resections are associated with less invasive incision sizes, less postoperative ileus and earlier tolerance of diet which may contribute to less need for analgesic treatment and earlier recovery of the patient with a reduced hospital stay. A faster hospital recovery has been demonstrated to translate significantly lower total costs owing to lower pharmacy, laboratory, and ward nursing costs. Reduced analgesia requirements and lower occurrence of complications may also decrease costs associated with laparoscopic treatment. However, studies reporting differences between the two procedures are equivocal.

The most recent reports concluded that laparoscopic colorectal resections are significantly cheaper than conventional open resections because of the reduced hospital stay, despite higher operative spending. The average reduction in total direct costs is difficult to define due to the increasing cost over time making comparisons between studies conducted during a time range of more than 10 years. Moreover, the costs may vary according to region or country in which the study was performed. The saving cost estimated per case has been reported to range from about $50 to $500. However, the analysis of the source of such costs demonstrates that this reduction is derived mostly from nursing care, pharmacy, and laboratory costs which compensate the increased operating room expenses incurred by laparoscopic surgery. On the other hand, newly designed "fast-track" care for colectomy patients may narrow the distinctions between hospital stay of laparoscopic and open colectomy because of the perception that length of stay can be dramatically reduced with open surgery [72-75].

## Conclusions

The laparoscopic approach to colectomy is slowly gaining acceptance for the management of colorectal pathology. Laparoscopic surgery for colon cancer demonstrates better short-term outcome, oncologic safety, and equivalent long-term outcome of open surgery. For rectal cancer, laparoscopic technique can be more complex depending on the tumor location. However, improvements in health outcomes have been reported also for rectal location of cancers, with comparable results to open surgery when the experience of the surgeon is well established. The advantages of minimally invasive surgery may translate better care quality for oncological patients and lead to increased cost saving through the introduction of active enhanced recovery programs which are likely cost-effective from the perspective of the hospital health-care providers.

## Competing interests

The authors declare that they have no competing interests.

## Authors' contributions

AB: conception and design, drafting the manuscript; GG, AM, SM, CT: drafting the manuscript; FD, SG, FB: critical revision, given final approval of the version to be published.

## Declarations

Funding of this article came from University funds.

This article has been published as part of *BMC Surgery *Volume 13 Supplement 2, 2013: Proceedings from the 26th National Congress of the Italian Society of Geriatric Surgery. The full contents of the supplement are available online at http://www.biomedcentral.com/bmcsurg/supplements/13/S2
